# Effects of probiotic and metformin co-administration versus metformin monotherapy on anthropometric measurements, hormones, and glucolipid profile in women with polycystic ovary syndrome: a systematic review and meta-analysis

**DOI:** 10.3389/fendo.2026.1802369

**Published:** 2026-03-26

**Authors:** Mohammed Hamsho, Wijdan Shkorfu, Meriem Bensaoua, Carlos Eduardo Carvalho Martins, Hale Hacıbayram, Abdulmannan Fadel, Yazan Ranneh

**Affiliations:** 1 Department of Nutrition and Dietetics, Faculty of Health Science, Istanbul Yeni Yuzyil University, Istanbul, Türkiye; 2 Department of Nutrition and Dietetics, Faculty of Health Sciences, Bahcesehir University, Istanbul, Türkiye; 3 Department of Nutrition and Dietetics, Faculty of Health Sciences, Istinye University, Istanbul, Türkiye; 4 Faculty of Medicine, University of São Paulo, São Paulo, Brazil; 5 Department of Nutrition and Health, College of Medicine and Health Sciences, United Arab Emirates, Al Ain, United Arab Emirates; 6 Department of Nutrition and Dietetics, College of Pharmacy, Al-Ain University, Abu Dhabi, United Arab Emirates

**Keywords:** combination, insulin resistance, meta-analysis, metformin, PCOS, polycystic ovary syndrome, probiotic

## Abstract

**Background:**

Polycystic ovary syndrome (PCOS) often presents with insulin resistance and hormonal imbalances, metformin and probiotics are each effective treatments but the effect of combining both is unknown.

**Objective:**

To assess whether the addition of probiotics to metformin treatment further improves metabolic, hormonal and gastrointestinal effects in women with PCOS. Methods: PubMed, Web of Science, Scopus, and Google Scholar were searched for English-language articles until 2 December 2025. Two reviewers screened studies, extracted data and evaluated risk of bias independently. Random effects pooled effects were calculated when possible as mean difference (MD) or standardized mean difference (SMD) with 95% confidence intervals (CI).

**Results:**

Six studies were included; compared to metformin alone the addition of probiotics decreased insulin resistance (homeostasis model assessment of insulin resistance (HOMA-IR): MD -0.50, 95% CI -0.73 to -0.26; low-to-moderate heterogeneity) and borderline significantly lowered luteinizing hormone (LH) (SMD -0.56, 95% CI -1.11 to 0.01; substantial heterogeneity), there were also fewer metformin-induced gastrointestinal adverse effects with the addition of probiotics.

**Conclusions:**

There may be benefit to adding probiotics to metformin therapy for women with PCOS in improving insulin resistance and gastrointestinal tolerability as well as possibly decreasing LH. Heterogeneity between studies, short intervention duration and non-standardized probiotic doses/preparations limit the strength of these findings. Further study with larger, longer, randomized controlled trials that use a standardized probiotic formulation are needed.

**Systematic review registration:**

https://www.crd.york.ac.uk/PROSPERO/view/CRD420251237018, identifier CRD420251237018.

## Introduction

Polycystic Ovary Syndrome (PCOS) is an ovarian dysfunction syndrome. Its principal defining characteristics are hyperandrogenism and polycystic ovarian morphology (1). Clinically, the syndrome manifests symptoms such as obesity, clinical signs of androgen excess, and irregular menstruation; and is also associated with an increased incidence of type 2 diabetes (T2D) (2–4).

The diagnosis of this syndrome is established based on the clinical and laboratory criteria defined in the “International Evidence-Based Guideline for the Assessment and Management of PCOS 2023.” Most medical society guidelines accept that PCOS can be diagnosed if two out of the three following clinical criteria, known as the Rotterdam Criteria, are present: hyperandrogenism (clinical, manifested by hirsutism, acne, or hair loss, or biochemical), polycystic ovarian morphology (demonstrated by multiple follicles in the ovary via ultrasound or by elevated anti-Müllerian hormone (AMH) levels), and ovarian dysfunction/anovulation (characterized by the absence of or irregular menstruation). It is crucial to note that no single test is sufficient to confirm or exclude the diagnosis of PCOS (5).

In PCOS, insulin resistance is a central driver of both hyperandrogenism and ovulatory dysfunction. The resulting hyperinsulinemia enhances androgen production by theca cells, disrupts the activin–inhibin–follistatin axis, and impairs follicle sensitivity to Follicle-Stimulating Hormone (FSH), thereby hindering normal follicular development (6). In parallel, elevated insulin and Insulin-like Growth Factor 1 (IGF-1) amplify Luteinizing Hormone (LH) signaling, further obstructing follicular maturation and promoting chronic anovulation. Together, these mechanisms shape the clinical phenotype of PCOS and underscore the importance of therapeutic strategies aimed at improving insulin sensitivity (7, 8).

Gut microbiota dysbiosis in PCOS patients leads to increased intestinal permeability, allowing microbial compounds to enter systemic circulation, resulting in chronic systemic inflammation that contributes to insulin resistance, hyperandrogenism, and mood disorders; hallmarks of the condition (9). A decline in beneficial bacteria like *Bifidobacteria* and *Lactobacillus*, which normally maintain gut health, can exacerbate this process, further contributing to the development of PCOS (10).

Given the established link between gut dysbiosis and PCOS pathophysiology, metformin treatment is hypothesized to exert part of its therapeutic effect through microbiota. Metformin treatment modulates the gut microbiota by significantly increasing the abundance of short-chain fatty acid (SCFA)-producing bacteria, including *Akkermansia*, *Lactobacillus*, *Bifidobacterium*, *Prevotella*, *Megasphaera*, *Shewanella*, *Blautia*, and *Butyrivibrio* (11, 12). Concurrently, it can decrease the presence of harmful bacteria, such as *Clostridium perfringens* and *Clostridium difficile* (13). Metformin’s modulatory effect may also stem from the activation of intestinal adenosine monophosphate kinase (AMPK), which subsequently influences the secretion of antimicrobial peptides and affects the overall homeostatic balance of the gut microbiota (14).

Building upon the evidence that metformin modulates the gut, probiotic supplementation represents a direct therapeutic approach to correct dysbiosis. Probiotics have shown clinically significant improvement in homeostatic model assessment of insulin resistance (HOMA-IR) in adults with T2D (15). Similarly, probiotic supplementation significantly improved fasting blood glucose, insulin resistance, and c-reactive protein levels in women with PCOS (16).

Conversely, given that Metformin fundamentally improves insulin sensitivity, the co-administration of probiotics presents a promising therapeutic strategy. It is hypothesized that probiotics could amplify Metformin’s beneficial metabolic effects by actively restoring a healthy gut microbial balance, reducing systemic inflammation, and alleviating GI symptoms (17). Despite the potential synergistic interaction between metformin and probiotics, there is no published meta-analysis investigating this issue.

Consequently, there is a need for a quantitative synthesis of the available evidence. By using rigorous methods such as meta-analysis, this study aims to compare the efficacy and safety between probiotic and metformin co-administration to metformin monotherapy in women with PCOS on anthropometric measurements, metabolic profile, sex hormones, and side effects.

## Methods

We performed a systematic review based on the guidelines of the Preferred Reporting Items for Systematic Reviews and Meta-Analyses (PRISMA) (18). The study protocol was registered in PROSPERO with the following ID: CRD420251237018.

### Search strategy

A search of online databases including PubMed, Web of Science, and Scopus was conducted up to the cut-off date of the 2nd of December 2025 by two independent authors (M.T. and A.F.). In addition, the first 59 results of Google scholar were obtained. We systematically searched the literature to identify human studies that compared between probiotic and metformin co-administration to metformin alone. A comprehensive search was conducted to identify relevant studies by examining electronic databases using relevant Medical Subject Headings (MeSH) (**Supplementary Table 1**). In addition to database searching, the reference lists of all included articles were manually screened for additional eligible studies.

### Eligibility criteria

Studies were included if they met the following criteria: (1) included human adult women diagnosed with PCOS; (2) investigated the combined impact of single or multiple strains probiotic + metformin for at least 1 month; (3) utilized metformin as comparator; (4) evaluated at least one of the following outcomes: anthropometric indices; glucose and lipid metabolism–related parameters; or circulating sex hormones; (5) were either experimental or observational study, peer-reviewed, published as full-text articles in the English language.

Conversely, studies were excluded if they were if they: (1) were animal or invitro studies, case reports, literature reviews, meta-analyses, conference abstracts, letters, or editorials; (2) utilized different metformin doses between the groups; (3) lack statistical information to be included in the forest plot.

### Study selection

Study selection was performed independently and in duplicate by two reviewers (M.H. and M.B.). In the first stage, titles and abstracts of all retrieved records were screened according to the predefined eligibility criteria. In the second stage, the full texts of potentially eligible articles were assessed in detail against the inclusion and exclusion criteria. Any discrepancies between reviewers were resolved through discussion or, when necessary, by consultation with a third senior investigator (Y.R.). The study selection process was documented using a PRISMA flow diagram, and reasons for exclusion were recorded at each stage.

### Data extraction

After the selection process of articles, with regard to the eligibility criteria, characteristics of included studies information including (study, country, study sample size (intervention/control), age (mean ± standard deviation (SD)), intervention, control, and duration were extracted by two independent authors (M.H. and H.H.) (**Table 1**).

**Table 1:** Characteristics of included studies.

Author	Study design	Country	Sample size I/C	Age (mean ± SD)	Intervention	Control	Duration
(23)	RCT	Pakistan	52 (26, 26)	Combined group: (25.1 ± 5.3) Metformin group: (27.2 ± 4.6)	Probiotic 180 mg/day, containing (Lactobacillus Acidophilus (1 X 109 CFU/g), Lactobacillus Delbruekii (1 X 109 CFU/g), Bifidobacterium Bifidum (1 X 109 CFU/g) Lactobacillus Bulgaricus and Streptococcus Thermophilus) + Metformin 500 mg twice daily	Metformin 500 mg twice daily	3 months
(24)	RCT	China	40 (20, 20)	24.73 ± 4.91 years	Probiotics power (4 g daily) + Metformin (1.5 g daily)	Metformin 500 mg, three times a day	3 months
(25)	RCT	China	96 (49, 47)	Combined group: (27.17 ± 5.58) Metformin group (27.43 ± 5.40)	*L. acidophilus* JYLA-126+ metformin 500 mg twice daily	Metformin 500 mg twice daily + placebo (maltodextrin 1 sachet three times daily)	3 moths
(26)	RCT	Bangladesh	60 (30, 30)	Combined group: (24.5 ± 3.1) Metformin group: (24.2 ± 3.6)	Probiotic capsules containing Lactobacillus spp. and Bifidobacterium spp. 4 billion CFU twice daily for 12 weeks + metformin 500 mg once daily for 7 days, then twice daily for the next 7 days and thrice daily for the remaining 10 weeks.	Metformin in the same dosage regimen.	3 months
(27)	Retrospective observational cohort study	China	138 (68, 70)	Combined group: (28.68 ± 6.45) Metformin group: (28.53 ± 6.24)	one sachet of probiotics powder 2 times a day + Metformin 500 mg/3 times a day	Metformin 500 mg/3 times a day	3 months
(28)	Retrospective observational cohort study	China	141 (72, 69)	Combined group: (31.53 ± 5.52) Metformin group: (30.67 ± 5.867)	Bifidobacterium Triple Viable Capsules (0.21g/capsule) were orally taken at a dose of 0.84g/time, twice a day, continuously + Metformin 500 mg once daily for 7 days, then twice daily for the next 7 days and thrice daily for the remaining 10 weeks.	Metformin in the same dosage regimen.	3 months

### Risk of bias assessment

The quality of the included randomized controlled trials (RCTs) was evaluated according to Cochrane Collaboration’s tool (19). This tool has the following key parts: (1) randomization process, (2) deviations from intended interventions, (3) missing outcome data, (4) measurement of the outcome, (5) and selection of the reported results. Each item was categorized as having a low risk of bias, some concerns, and high risk of bias. Accordingly, the overall risk of bias was determined based on the highest risk of bias across the domains.

The ROBINS-I V2 (Risk Of Bias In Non-randomized Studies – of Interventions, Version 2) was used to assess the risk of bias of non-randomized trials. This tool has the following key parts: (1) confounding, (2) classification of intervention, (3) selection into the study, (4) missing data, (5) measurement of the outcome, (6) and selection of reported results. Accordingly, each domain was categorized as having a low/moderate/serious/critical risk of bias. The overall risk of bias for each study was determined by the highest risk of bias attributed to any individual domain, in accordance with ROBINS-I guidance (20). All studies selected for retrieval were assessed by two independent reviewers (M.H. and Y.R.). Discrepancies in assessments were resolved through discussion.

### Statistical analysis

This meta-analysis was performed using the Cochrane Program Review Manager Version 5.4. Variables assessed in at least three studies or more with sufficient quantitative data were included. Variables reported in fewer than three studies were synthesized qualitatively. In this regard, pre- and post-values of the mean ± SD of fasting blood glucose (FBG), fasting insulin (FINS), homeostatic model assessment of insulin resistance (HOMA-IR), body mass index (BMI), serum luteinizing hormone (LH), serum Follicle-stimulating hormone (FSH), serum Testosterone, high-density lipoprotein cholesterol (HDL-C), triglyceride (TG) were assessed. If the median with Q1 and Q3 was reported, the data were converted to mean and SD based on the method described here (21).

When change of SD was not given, SDs of mean differences were calculated by using the following equation: ${\text{SD}}_{E,\text{change}}=\sqrt{{\text{SD}}_{E,\text{baseline}}^{2}+{\text{SD}}_{E,\text{final}}^{2}-\left(2\times \text{Corr}\times {\text{SD}}_{E,\text{baseline}}\times {\text{SD}}_{E,\text{final}}\right)}$ where the correlation coefficient (R) was assumed to be 0.8 (22). In order to apply mean difference in forest plots, units were converted into one measurement by using Unitslab and Laborteam when applicable prior to performing the analysis. However, the conversion of certain outcomes was not feasible due to the lack of a universally accepted conversion between these unites (mIU/mL and pmol/L), therefore we analyzed these outcomes using standardized mean difference (SMD) with 95% CIs and mean differences (MD) with 95% CIs for the rest of the outcomes. The random-effects model was applied for pooling analysis to compensate for the heterogeneity of the studies (22). Interstudy heterogeneity was explored quantitatively using Cochran’s Q and I2 statistics. I2 ≤ 50% and ≥75% indicated substantial and considerable heterogeneity, respectively (22). P-values were considered statistically significant at< 0.05.

## Results

### Literature selection

**Figure 1:**
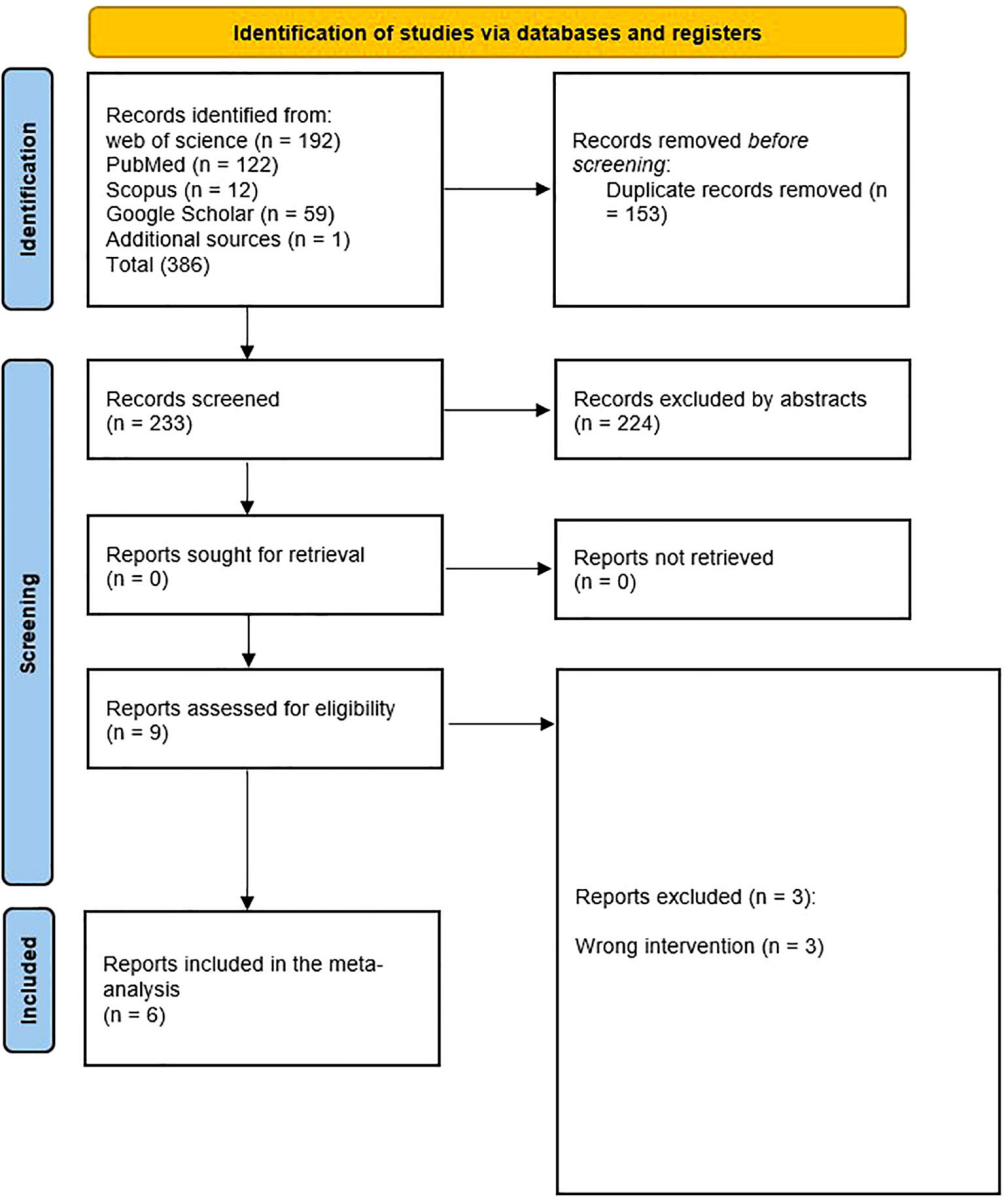
Prisma flow chart.

Based on the selection criteria set for this meta-analysis, 386 articles were identified through initial search in databases. The focus was on the interventional studies assessing the added effect of probiotics to metformin compared to metformin alone in managing dysregulated metabolic and hormonal symptoms in women with PCOS. Duplicate references (n = 153) were excluded, and (n = 224) articles were eliminated by the titles or abstracts, and (n = 9) articles were eligible for inclusion in the systematic review and meta-analysis. Finally, (n = 6) were included in the quantitative and qualitative analysis (**Figure 1**).

### Studies’ characteristics

Six studies consisted of 527 women diagnosed with PCOS according to Rotterdam criteria. The duration of the included studies was 12 weeks. Studies were conducted various geographical locations China (n = 4) (24, 25, 27, 28) Pakistan (n = 1) (23), and Bangladesh (n = 1) (26). Four studies utilized RCT study design (23–26), and two were retrospective cohort studies (27, 28). Control groups administered metformin with a dose ranging from 1000 to 1500 mg/day. Interventional groups consumed the same metformin dose along with single or multi strain probiotic supplementation. *Lactobacillus* was utilized in one study (25), *Bifidobacterium* in one study (28). *Lactobacillus* and *bifidobacterium* combination in two studies (23, 26), and two studies didn’t specify any taxonomy (24, 27).

### Risk of bias

**Figure 2:**
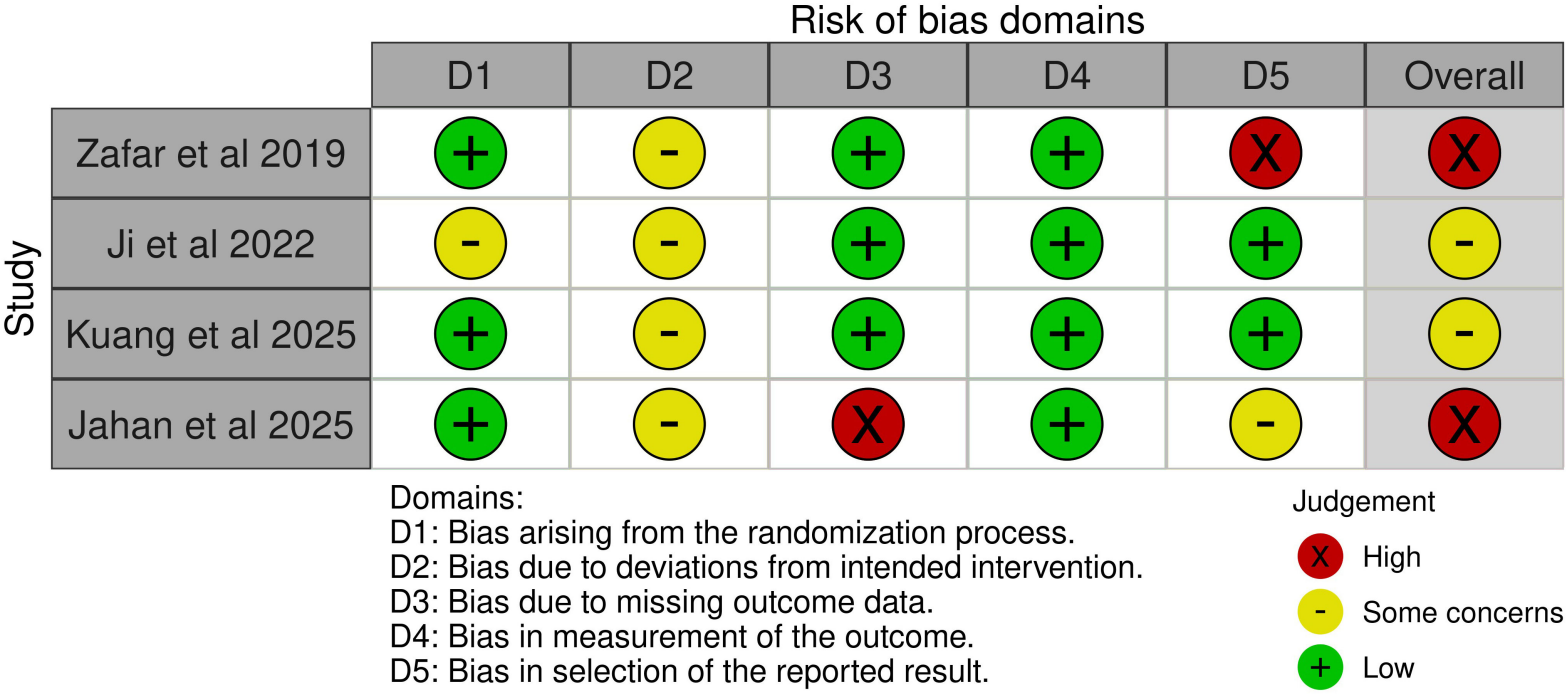
ROB-2 assessment of the included RCTs.

**Figure 3:**
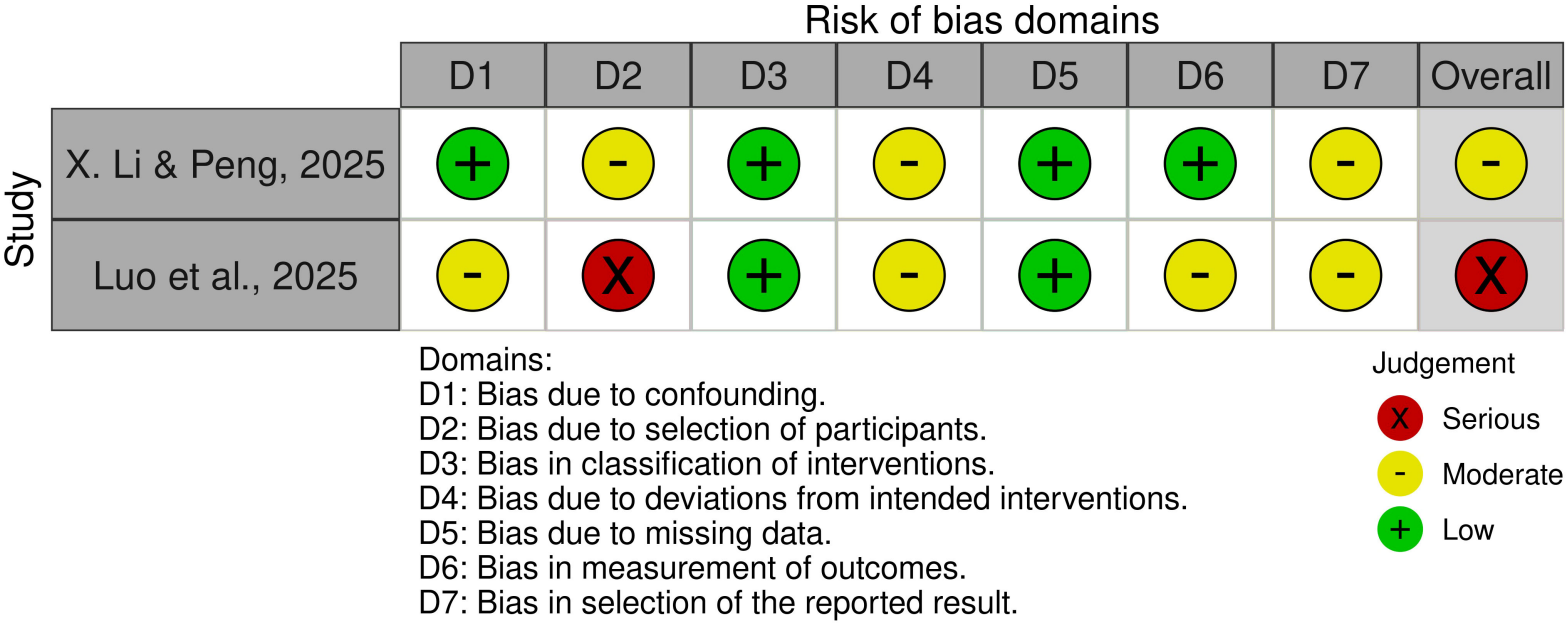
ROBINS-I assessment of the included non-randomized trials.

Among the four RCTs, two studies were judged to have an overall high risk of bias, while the remaining two were assessed as having some concerns (**Figure 2**). For the non-randomized trials, one study was judged to have a moderate risk of bias, whereas the other was assessed as having a serious risk of bias (**Figure 3**).

### Meta-analysis

#### Effects of probiotic supplementation with metformin on metabolic profile

**Figure 4:**
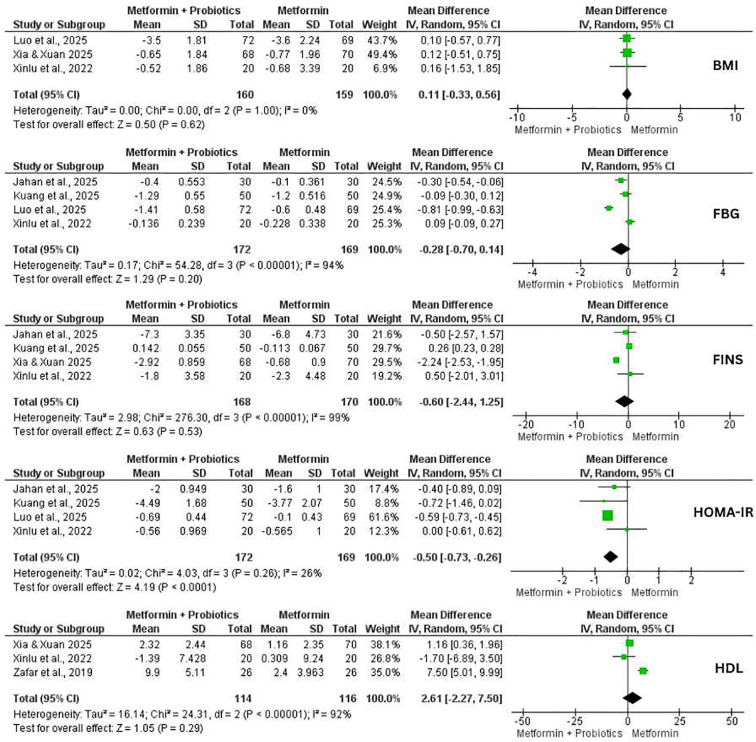
Forest plots for body mass index (BMI), fasting blood glucose (FBG), fasting Insulin (FINS), homeostatic model assessment of insulin resistance (HOMA-IR), and high-density lipoprotein cholesterol (HDL-C) of metformin combined with probiotics vs. metformin alone for women with polycystic ovarian syndrome (PCOS).

Three studies evaluated the effect of probiotics co-administration with metformin on anthropometric changes, and four studies assessed the metabolic profile in women with PCOS (**Figure 4**). Most pooled outcomes did not demonstrate statistical significance differences between groups. Although effect estimates favored the combination in general, confidence intervales were wide and heterogeneity was considerable. There was no statistical difference in BMI (MD = 0.11, 95% CI: -0.33, 0.56, p = 0.62, I^2^ = 0), and FBG (MD = -0.28, 95% CI: -0.70, 0.14, p = 0.20, I^2^ = 95%) between intervention and control groups (**Figure 4**). Moreover, the pooled analysis demonstrated statistically insignificant differences in FINS (MD = -0.60, 95% CI: -2.44, 1.25, p = 0.53, I^2^ = 99%), and HDL-C (MD = 2.61 mg/dL, 95% CI: -2.27, 7.50, p = 0.29, I^2^ = 92%). However, there was a significant reduction in HOMA-IR in the intervention group supplementing probiotics with metformin compared to metformin alone (MD = -0.50, 95% CI: -0.73, -0.26, p< 0.0001, I^2^ = 26%).

#### Effects of probiotic supplementation with metformin on sex hormones

**Figure 5:**
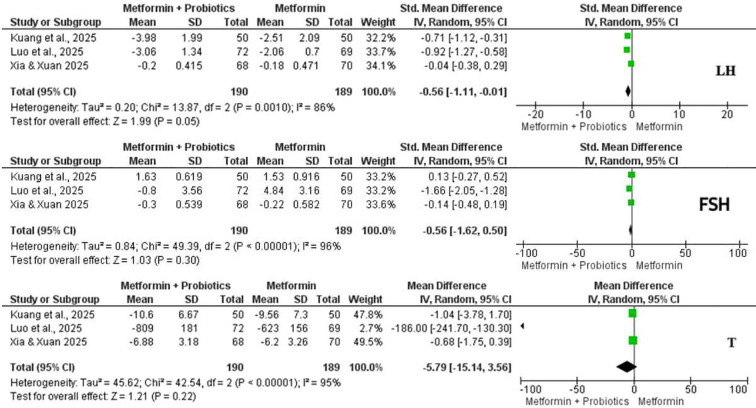
Forest plots for serum luteinizing hormone (LH), follicle-stimulating hormone (FSH), and serum testosterone (T) of metformin combined with probiotics vs. metformin alone for women with polycystic ovarian syndrome (PCOS).

Three studies examined the effect of the combination treatment on sex hormones (**Figure 5**). LH considerably reduced following intervention with high heterogeneity (SMD = -0.56, 95% CI: -1.11, 0.01, p = 0.05, I^2^ = 86%). On the other hand, there was a non-significant trend toward reduction in testosterone (MD = -5.79, 95% CI: -15.14, 3.56, p = 0.22, I^2^ = 95%) and FSH at the end of the study (SMD = -0.56, 95% CI: -1.62, 0.50, p = 0.30, I^2^ = 96%) (**Figure 5**).

### Qualitative synthesis

#### Effects of probiotic supplementation with metformin on anthropometrics

In the study investigated by Ji et al. (24), both intervention (n = 20) and control group (n = 20) demonstrated significant improvements in BW (kg) from baseline. Control group (MD = - 4.40 ± 11.7 kg, p< 0.01) exhibited greater mean reduction in BW (kg) compared to the intervention group (MD = -1.51 kg ± 7.3 kg, p< 0.01); however, between group statistical significance could not be assessed. Similarly, Zafar et al. (23) reported significant within-group reduction in BW (kg) in both groups (p< 0.01). However, intervention group (n = 26) showed greater change in magnitude compared to control group (n = 26) (MD = -5 ± 10.8 kg) (MD = -3.1 ± 9.77 kg), respectively.

A total of two studies assessed the effect of supplementing probiotics with metformin in WC (cm). Li and Peng (27) reported significant differences between combination group (n = 68) (MD = -7.35 ± 3.61 cm), and metformin group (n = 70) (MD = -10.2 ± 3.1 cm) (p= 0.037). Moreover, Ji et al. (24) indicated a significant within-group reduction in WC in both, control group (n = 20) (MD = - 3.12 ± 6.8 cm, p< 0.01) and intervention group (n = 20) (MD = -1.97 ± 5.89 cm, p< 0.05) compared to baseline; however, between group statistical significance could not be assessed.

Furthermore, only one study reported data on HC (24). There was a non-significant trend towards reduction in HC in intervention group (MD = -1.08 ± 5.19 cm) and control group (MD = -2.1 ± 5.2 cm).

#### Effects of probiotic supplementation with metformin on lipid profile

For LDL-C, two studies provided within-group data on this outcome. Ji et al. (24) reported a significant reduction in LDL-C in control group (MD = -3.25 ± 19.5 mg/dL) indicating a greater change compared to intervention group (MD = 1.39 ± 22.6 mg/dL). Conversely, Zafar et al. (23) reported a statistically significant reduction in the intervention group (MD = -3.8 ± 9.07, p< 0.01) relative to control group (MD = -1.1 ± 9.21 mg/dL).

Similarly, conflicting results were reported by two studies on TC. Ji et al. (24) reported a significant reduction in TC in control group (MD= -19.6 ± 35.3, p< 0.05) opposite to intervention group (MD = -4.95 ± 24.1). While Zafar et al. (23) demonstrated a statistically significant within-group reduction in intervention group (MD = -13.3 ± 13.9, p< 0.01) and control group (MD = -9.4 ± 13.3, p< 0.01).

Regarding TG,Ji et al. (24), the metformin group showed a significant decrease in TG (MD = -6.65 ± 17.6 mg/dL) (p< 0.05) when compared to the combination group (MD = 1.39 ± 20.5 mg/dL). Besides, Zafar et al. (23) indicated a significant reduction in control (MD = -6.3 ± 11.52 mg/dL, p< 0.01) and intervention groups (-9.9 ± 11.2, p< 0.01).

### Side effects

Jahan et al. (26) reported a lower prevalence of gastrointestinal symptoms in the combination group compared with the metformin group, including bloating (9.1% vs. 29.2%), nausea (13.6% vs. 25%), and diarrhea (0% vs. 8.3%). Similarly, Kuang et al. observed a significantly lower prevalence of epigastric pain, nausea, acid regurgitation, and diarrhea among participants receiving the combination therapy. Ji et al. (24) found that in the metformin group, nausea, diarrhea, and constipation were reported by 25%, 80%, and 10% of patients, respectively, whereas in the combination group, nausea and diarrhea occurred in 10% of participants, with diarrhea reported by 30% (n = 6) and nausea and constipation each reported by 5% (n = 1).

## Discussion

To our knowledge, this is the first systematic review and meta-analysis investigates the effects of probiotic supplementation and metformin co-administration compared to metformin monotherapy in women with PCOS. Our results demonstrate that the combination significantly improved insulin resistance, as assessed by HOMA-IR, and reduced GI complications as well as considerable reduction in LH levels compared with metformin alone. However, no significant effects were observed on anthropometric measures, lipid profiles, glycemic indices, or sex hormone levels. Collectively, these findings suggest that probiotic supplementation may enhance specific metabolic and tolerability-related outcomes when used as an adjunct to metformin, rather than exerting broad metabolic or endocrine effects in women with PCOS.

Insulin resistance serves as the main contributor to androgen hormones production in PCOS, through affecting gonadotropin secretion and ovarian steroidogenesis. HOMA-IR is widely accepted as insulin resistance indicator, correlates higher values with disease severity (25, 29). The greater reduction in HOMA-IR observed with probiotic and metformin combination therapy may underlie the associated decrease in LH levels, as hyperinsulinemia has been shown to stimulate pituitary LH secretion and enhance ovarian androgen production. These findings support the hypothesis that ameliorating insulin resistance may indirectly modulate hormone dysregulation in PCOS women (30).

Although metformin is used as first line treatment of insulin resistance in PCOS, higher doses are associated with gastrointestinal discomforts such as nausea, diarrhea, constipation, and bloating. These adverse effects often limit treatment adherence, highlighting the need for adjuvant therapies that improve drug tolerability and potentially exert additional health benefits (17, 31). Our results indicate that combination group had significantly lower odds of GI adverse effects compared to metformin alone group.

Emerging evidence highlights the role of gut microbiota dysbiosis to PCOS pathogenesis, mainly due to systematic inflammation, increased intestinal permeability, and disruption of lipid and bile acid metabolism along with reduction of alpha diversity and depletion of beneficial taxa (9, 32). Accordingly, probiotic supplementation is hypothesized to partially compensate for the function of the non-existed health enhancing microbes, specifically by production of SCFA, improving intestinal integrity, reduction of pro-inflammatory cytokines, and increasing beneficial and inhibiting pathogenic bacteria in the colon (17). Collectively, these mechanisms may act synergistically with metformin, resulting in improved insulin signaling and enhanced insulin sensitivity in peripheral tissues.

To date, only one meta-analysis compared between probiotic and metformin combination versus metformin alone. Consistent with our findings, there was a reduction in insulin resistance along with improvement in GI side effects in combination group in T2D (33).

Despite improvements in insulin resistance and LH levels, combining probiotic supplementation with metformin did not result in significant changes in body weight, body mass index, lipid profiles, glycemic indices, or most sex hormone concentrations. These null findings may be attributed to the relatively short duration of the included interventions, which may be insufficient to induce measurable changes in sex hormones. Therefore, future longitudinal trials are warranted.

From a clinical perspective, probiotic supplementation may be advised for women with PCOS as adjuvant therapy with metformin, particularly those experiencing abnormal levels of insulin resistance and GI symptoms.

## Limitations and future directions

While the findings are promising for management of PCOS, our meta-analysis contains several limitations that must be acknowledged. First, the number of available studies comparing probiotic and metformin co-administration to metformin in PCOS was limited, resulting in relatively small pooled sample size, which reduced statistical power and may have limited the ability to detect significant effects.

Second, variability in the methodological quality and design of the included studies represents an important limitation. Some trials provided insufficient detail regarding randomization procedures, allocation concealment, blinding, and selective reporting which may increase the risk of bias and affect the reliability of pooled estimates.

Third, one of the most concerning aspects of interventions across the trials is the lack of clear description of probiotic taxa and amount of viable microorganisms. On the other hand, some studies utilized single strains, whereas the others utilized multi-strains probiotic, which may explain the heterogeneity and discrepancy among the studies These factors limit the comparability of findings across trials and precludes conclusions regarding strain-specific efficacy or underlying mechanisms.

Fourth, the duration of the included studies was relatively short to detect changes in sex hormone levels. Given that metformin alone induces gradual improvements in body weight, hormonal profiles, and reproductive outcomes, longer intervention periods may be required to observe any additional or minimal effects attributable to probiotic supplementation. Consequently, the limited study duration may have contributed to the small or nonsignificant effects observed for hormonal outcomes.

Given these limitations, future RCTs with larger sample sizes, longer follow-up durations, and standardized probiotic formulations are needed to confirm these findings. Studies stratifying participants by PCOS phenotype may further clarify underlying mechanisms and help identify subgroups most likely to respond to probiotic and metformin co-administration.

## Conclusions

Combination of probiotic supplementation and metformin appears to reduce insulin resistance, luteinizing hormone, and alleviate gastrointestinal symptoms associated with metformin administration in women with PCOS. In contrast, its effects on anthropometric measurements, lipid profile, sex hormones were minimal. These preliminary findings should be interpreted with caution, as they were driven from low number of studies with limited sample size and heterogeneous probiotic formulations.

## Data Availability

The original contributions presented in the study are included in the article/**Supplementary Material**. Further inquiries can be directed to the corresponding author.
