# Supplementary material for: Effects of probiotic and metformin co-administration versus metformin monotherapy on anthropometric measurements, hormones, and glucolipid profile in women with polycystic ovary syndrome: a systematic review and meta-analysis
Source: Front Endocrinol (Lausanne). 2026 Mar 26;17:1802369. doi: 10.3389/fendo.2026.1802369 (PMC13061675; doi:10.3389/fendo.2026.1802369)
Supplement: Supplementary file 1 [file Table1.docx]

**Table S1: Electronic search strategies**

| **Database** | **Search Strategy** | **N** |
| --- | --- | --- |
| **PubMed** | ("pcos"[Title/Abstract] OR "polycystic ovarian syndrome"[Title/Abstract] OR "polycystic ovary syndrome"[Title/Abstract]) AND ("probiotic"[Title/Abstract] OR "lactobacillus"[Title/Abstract] OR "bifidobacterium"[Title/Abstract] OR "lactococcus"[Title/Abstract] OR "single strain"[Title/Abstract] OR "multi strain"[Title/Abstract]) | 122 |
| **Scopus** | TITLE-ABS-KEY(PCOS OR polycystic ovary syndrome OR polycystic ovarian syndrome) AND TITLE-ABS-KEY (probiotic OR lactobacillus OR bifidobacterium OR Lactococcus OR single strain OR multi strain) | 12 |
| **Web of Science** | 1. TS=(kefir OR "fermented milk") 2. AND TS=("gut microbiota" OR "gut microflora" OR "intestinal microbiota" OR "gastrointestinal microbiota") 3. AND TS=("clinical trial" OR "randomized controlled trial" OR intervention OR "interventional study") 4. AND TS=(human OR humans OR adult) | 192 |
| **Google scholar** | (pcos OR polycystic ovary syndrome OR polycystic ovarian syndrome) AND (probiotic OR lactobacillus OR bifidobacterium OR lactococcus OR single strain OR multiple strain) AND (metformin) | 59 |
